# Incidence and characteristic analysis of in-hospital falls after anesthesia

**DOI:** 10.1186/s13741-016-0038-z

**Published:** 2016-05-23

**Authors:** Chen-Fuh Lam, Shiu-Ying Hsieh, Jen-Hung Wang, Hui-Shan Pan, Xiu-Zhu Liu, Yu-Ching Ho, Tsung-Ying Chen

**Affiliations:** Department of Anesthesiology, Buddhist Tzu Chi General Hospital and Tzu Chi University School of Medicine, No 707, Chung-Yang Road Section 3, Hualien, 970 Taiwan Republic of China; Department of Medical Research, Tzu Chi General Hospital, Hualien, Taiwan; Department of Health Administration, Tzu Chi University of Science and Technology, Hualien, Taiwan

**Keywords:** Accidental falls, Poisson distribution, Postoperative complications, Regional anesthesia

## Abstract

**Background:**

In-hospital falls may result in serious clinical adverse consequences, but the effects of anesthesia in the occurrence of postoperative falls are still undetermined. Anesthesia may theoretically cause postoperative falls due to the residual pharmacologic and neuromuscular blocking effects of anesthetics. We retrospectively reviewed events of in-hospital falls occurred after anesthesia management to identify the incidence and risk factors of postanesthesia falls.

**Methods:**

We reviewed the postanesthesia visit of patients received anesthesia in the Hualien Buddhist Tzu Chi General Hospital from January 2009 to December 2013. Falls happened within 24 h after anesthesia were recorded. The Poisson regression model was used for simultaneous analysis of the association between incidence proportion of postanesthesia falls and the potential risk factors.

**Results:**

A total of 60,796 inpatients received anesthesia management over the past 5 years, and ten patients fell within 24 h after anesthesia. All cases happened in the general wards. Falls occurred more often at the bedside, presence of caregivers, and during the daytime. Patients underwent regional anesthesia, and old age significantly increased the risk of postanesthesia falls, while differences in gender and ASA physical status did not affect the occurrence of postanesthesia falls.

**Conclusions:**

The overall incidence proportion of postanesthesia falls is 1.6 cases per 10,000 patients (95 % CI 0.006 to 0.026 %) over a 24-h observation period. Falls are more commonly happened during the less expected periods after operation and are increased in the elderly and patients received regional anesthesia. This study highlights that more comprehensive clinical practice guidelines for postoperative care should be exercised to prevent the in-hospital falls.

## Background

Postoperative falls are a relatively rare complication that could have been overlooked in clinical anesthesia. In-hospital falls are particularly serious events resulting in bone fracture, traumatic head injury, visceral organ contusion, and skin abrasion. These in-hospital adverse events not only increase the length of hospital stay and cause extraneous medical expense but may also engender unnecessary medical disputes (Bates et al. 1995; Mion et al. 2012). Therefore, the implementation of preventive strategies for in-hospital falls has been an important practical issue in the hospital care (Hempel et al. 2013). However, the investigation of the in-hospital falls during the postoperative period is insufficient (Amador and Loera 2007; Church et al. 2011). The overall incidence of postoperative falls was 1.6–1.8 % in a veteran’s medical center with an average time to falling of 12 days after operation (Church et al. 2011). The incidence of postoperative falls may theoretically increase in patients receiving anesthetic management due to the residual pharmacologic and neuromuscular blocking effects of anesthetics. However, the anesthetic effect on the postoperative falls is hard to identify from these previous studies, and there is currently no study reported the incidence of postanesthesia falls. Therefore, we retrospectively reviewed the postanesthesia records (2009 to 2013) in our department and analyzed the characteristics of in-hospital falls after anesthesia management. Our long-term aim is to identify the risk factors contributing to falls during the postanesthesia period and to promote prevention programs in the surgical patients who carry high risk of in-hospital falls.

## Methods

### Patient database and study design

This retrospective chart-review study was approved by the ethics committee and the institutional review board (IRB, Approval number IRB103-14-B) of Hualien Buddhist Tzu Chi General Hospital Taiwan, and the requirement for written informed consent was waived by the ethics committee. Our hospital is a tertiary teaching medical center located at Hualien City of Taiwan that consists of 945 beds. In-hospital patients, who received anesthesia management for surgical or other medical interventions from January 2009 to December 2013, were visited at bedside postoperatively by nurse anesthetists. The postoperative visit recorded the pain scores, patient satisfaction to anesthesia service, and anesthesia-related complications. All adverse events after operation recorded by the ward nurses were reviewed. Falls were defined as sudden events which resulted in a person coming to rest inadvertently on the ground or floor or other lower levels (Haines et al. 2013). The medical records of all patients reported of having in-hospital falls within 24 h after operation were reviewed by the staff anesthesiologists to determine the characteristics of falls. Patients who received only regional infiltration of local anesthetics and who were discharged from hospital within 24 h after operation were excluded.

### Statistical analysis

The overall incidence proportion of postanesthesia falls over a 24-h observation period was calculated as the number of falls divided by total number of in-hospital surgical patients who received anesthesia management. The potential risk factors included patient demographic and clinical variables, namely patient age groups, genders, American Society of Anesthesiologists (ASA) classifications of physical status, and types of anesthesia. Poisson regression model was adopted to evaluate the association between these risk factors and postanesthesia falls. The model fitted reasonably well as the goodness-of-fit chi-squared test was not statistically significant (*p* = 0.995). Statistical significance was accepted at a level of *P* < 0.05. All statistical analyses were performed by using SAS 9.4 (SAS Institute, Inc., Cary, North Carolina).

## Results

### Patient characterization

From January 1, 2009 to December 31, 2013, there were a total of 78,064 individuals who received anesthesia management during surgical or other invasive interventions in the Hualien Buddhist Tzu Chi General Hospital, Taiwan, and 60,796 (77.9 %) of these patients were hospitalized for at least 24 h after anesthesia. The demographic characteristics of patients and types of operation are summarized in Table 1. The overall completion rate of in-hospital visit after anesthesia was 98.7 ± 0.5 % over the past 5 years and less than 0.5 % of missing data were found in our electronic data bank.

**Table 1:** Demographic characteristics of patients received anesthesia management during January 2009 to December 2013 (*n* = 78,064)

Patient characteristics	*N* (%)
Age (years old)	
0–10	2051 (2.6)
11–30	8409 (10.7)
31–50	23,143 (27.6)
51–70	31,226 (40.0)
>70	13,235 (16.9)
Gender	
M	39,941 (51.2)
F	38,105 (48.8)
ASA class^a^	
I	16,889 (21.6)
II	38,657 (49.5)
III	20,861 (26.7)
IV	1511 (1.9)
V	133 (0.2)
Emergency operation	3616 (4.7)
Type of anesthesia	
General anesthesia	57,619 (73.8)
Intravenous sedation anesthesia	18,100 (23.2)
Regional and other anesthesia techniques	2345 (3)
Admission after operation	
General wards	63,778 (81.7)
Intensive care units	7026 (9.0)
Discharged from recovery room	7182 (9.2)
Others	78 (0.1)
Completion of postoperative visits (in-hospital)	97.8 %

^a^A total of 13 patients were classed as ASA VI, who were admitted for organ transplant

### Postanesthesia falls

Events of falls within 24 h after anesthesia management were recorded in ten patients over the 5-year study period (Table 2). The incidence proportion of in-hospital falls after anesthesia was 0.016 % (95 % CI 0.006 to 0.026 %) over 24 h of observation. The average time of postoperative falls was 13.3 ± 8.4 (range 2.6–24) hours. All events of postanesthesia falls occurred at the general wards, as no falling events were reported from the special care. Table 3 illustrates the geographic and other characteristics of falling, including timing and location of falls. Most of these events were minor and did not result in prolonged hospital stay, but there were three victims who were more seriously injured who required extraneous medical or surgical attentions (Table 2).

**Table 2:** Characteristics of postanesthesia falls

Patient ID	Gender	Age (years)	ASA class	Type of surgery	Type of anesthesia	Injury after fall	Hours after anesthesia	Outcome
1	M	66	3	Debridement of hip wound	LMA	Scalp laceration	24	Wound repair
2	M	76	2	Herniorrhaphy	SA	NP	9.7	
3	F	64	2	Hemorrhoidectomy	IV sedation	NP	3.3	
4	M	61	2	FESS	ETGA	Scalp laceration	23.3	Wound repair
5	F	36	3	Arteriovenous graft	LMA	Head injury	19.8	Admitted to ICU
6	M	71	3	Endoscopic examination	IVG	NP	2.6	
7	F	52	1	Sling operation	SA	NP	8.5	
8	M	92	3	TURP	SA	Humeral fracture	23.5	ORIF
9	M	83	2	Endoscopic examination	IV sedation	Shoulder contusion	3.6	X-ray examination
10	F	53	2	Knee arthroplasty	LMA + NB	NP	14.6	

*FESS* functional endoscopic sinus surgery, *IV sedation* intravenous sedation anesthesia, *ICU* intensive care unit, *LMA* laryngeal mask anesthesia, *NB* femoral nerve block for pain control, *NP* nothing in particular, *ORIF* open reduction and internal fixation surgery, *SA* spinal anesthesia, *TUPP* transurethral resection of the prostate

**Table 3:** Characteristics of postanesthesia falls

Characteristics of fall events	*n* (%)
Time of fall	
Daytime (7:00–18:00)	7 (70)
Nighttime (18:01–06:59)	3 (30)
Place of fall	
Bedside	6 (60)
Washroom	4 (40)
Presence of accompany	
Yes	6 (60)
No	4 (40)
Attempts of getting off bed	
First	4 (40)
>1 attempts	6 (60)

Analysis of risk stratification indicated that gender and the ASA physical status of the patients with falls were not significantly different from the total and other in-hospital patients (Tables 4 and 5). However, age and types of anesthetic techniques were significant and independent risk factors for the occurrence of postanesthesia falls (Tables 4 and 5). Compared with younger ages, patients who were older (40–70, mean age of 65 ± 15 years) carried significantly higher incidence proportion of postanesthesia falls (*P* < 0.001). In addition, higher incidence proportions of postanesthesia falls were also found in patients who received regional anesthesia (neuroaxial blocks) and intravenous sedation anesthesia (*P* < 0.001 and *P* = 0.002, respectively).

**Table 4:** Risk stratification for postanesthesia falls

Patient characteristics	All patients (total 78,064) *n* (%)	In-hospital patients (total 60,796) *n* (%)	Postanesthesia falls (total 10) *n* (%)
Age (years old)			
0–10	2051 (2.6)	1385 (2.3)	0 (0)
11–30	8409 (10.7)	7544 (12.4)	0 (0)
31–50	23,143 (27.6)	17,498 (28.8)	1 (10)
51–70	31,226 (40.0)	22,594 (37.2)	5 (50)
>70	13,235 (16.9)	11,775 (19.3)	4 (40)
Gender			
Male	39,941 (51.2)	33,391 (54.9)	6 (60)
Female	38,105 (48.8)	27,405 (45.1)	4 (40)
ASA classification^a^			
I	16,889 (21.6)	9597 (15.8)	1 (10)
II	38,657 (49.5)	29,733 (46.0)	5 (50)
III	20,861 (26.7)	19,821 (32.6)	4 (40)
IV	1511 (1.9)	1500 (2.5)	0 (0)
V	133 (0.2)	132 (0.2)	0 (0)
Types of anesthesia			
General anesthesia	57,619 (73.8)	54,423 (89.5)	4 (40)
Intravenous sedation anesthesia	18,100 (23.2)	4057 (6.7)	3 (30)
Regional and other anesthesia techniques	2345 (3)	2316 (3.8)	3 (30)

^a^A total of 13 patients were classed as ASA VI, who were admitted for organ transplant

**Table 5:** Poisson regression analysis of the risk factors in the occurrence of postanesthesia falls

Risk factor	Regression coefficient	95 % CI	*P* value
Intercept	−34.434	(−36.158, −32.711)	<0.001
Gender			
Male	Ref	Ref	NA
Female	−0.012	(−1.303, 1.280)	0.986
Age			
≦30 years old	Ref	Ref	NA
31–50 years old	23.799	(21.489, 26.108)	<0.001
51–70 years old	25.408	(23.982, 26.834)	<0.001
>70 years old	25.555	(25.555, 25.555)	NA
ASA class			
I–II	Ref	Ref	NA
III–IV	−0.132	(−1.498, 1.235)	0.850
Anesthesia technique			
General anesthesia	Ref	Ref	NA
Intravenous sedation anesthesia	2.364	(0.853, 3.875)	0.002
Regional anesthesia	2.828	(1.267, 4.390)	<0.001

Reference groups were chosen according to the subgroup with highest proportion (i.e., male group, ASA class, and general anesthesia) or the first subgroup in the analysis

*CI* confidence interval, *Ref* reference value, *NA* not applicable

## Discussion

We present the first clinical report analyzing the incidence and risk factors of in-hospital falls within 24 h after anesthesia management. The overall incidence proportion of postanesthesia falls was at least 1.6 cases per 10,000 patients over a 24-h observation period. Patients with older age and who received regional anesthesia or intravenous sedation anesthesia carried significantly higher incidence proportion of postanesthesia falls. Falls happened more often during the relatively less expected periods and locations after operation, such as at the bedside, presence of caregivers, and during the daytime. Although most of these events did not result in serious consequences, there were considerable proportions of patients (30 %) who required additional medical or surgical attentions.

Successful surgical and anesthesia managements do not necessarily guarantee the avoidance of postoperative complications or adverse events. Therefore, several preoperative risk assessment systems have been developed to reduce the occurrence of postoperative adverse events (Barnett and Moonesinghe 2011; Hariharan and Zbar 2006). However, the identification of the incidence and risk factors of the rare adverse events is more challenging, as the conduction of prospective clinical studies is impractical and could be insensitive to detect the low incidence events. Therefore, the access to a comprehensive medical record of postoperative visit may be an acceptable alternative to prospective trials in the identification of the rare postoperative adverse events.

Postoperative falls are one of the relatively rare complications that could have been overlooked in clinical practice. There is currently very limited information in the study and prevention programs for postoperative falls (Church et al. 2011). Church et al. conducted a comprehensive retrospective study analyzing the occurrence of postoperative falls in patients admitted to a tertiary Veteran hospital for more than 23 h after surgery (Church et al. 2011). They reported that 154 male patients experienced 190 falls in a total of 9625 inpatient surgical procedures over the 5-year study period, thereby yielding an overall incidence of postoperative falls of 1.6 %. The authors found that older age, ASA class > III, low albumin level, longer surgical time, and increased blood transfusion were the independent risk factors for postoperative falls. Nevertheless, the effects of anesthesia on the occurrence of postoperative falls are still undetermined and are unable to generalize from these previous studies.

The Hualien Buddhist Tzu Chi General Hospital is a tertiary university hospital and admits all patient populations. The in-hospital postanesthesia visit recorded the anesthetic outcomes and complications detected within 24 h after anesthesia, with an average annual conclusion rate of greater than 97 % in 2009 to 2013. Therefore, the postanesthesia visiting records in our department may provide a reliable database for the analysis of the in-hospital adverse events that happened after anesthesia in the general population. All postoperative adverse events were reported by the ward nurses and our nurse anesthetists recorded the events occurred within 24 h after anesthesia, as the pharmacologic effects of anesthetic agents are theoretically diminished and most of the patients are recovered from the neuromuscular block.

A total of ten events of postanesthesia falls were recorded from 60,796 in-hospital anesthesia procedures during the 5-year study period. The incidence proportion of falls after operation was considerably less than that reported by Church et al. (0.016 vs 1.6 %). Obviously, the observational period of the later was longer (24 h vs 30 days postoperatively) and the later recruited surgical patients who received only local anesthesia. Contrarily, postanesthesia falls developed within 24 h after anesthesia resulted in much more serious injuries that required surgical or medical interventions (30 vs 0.3 %). These serious complications included traumatic head injury, bone fracture and ICU admission, and thus lengthening the in-hospital treatment course. Head-to-head comparison of the severity of outcomes after falling between these two studies is infeasible, since the studied populations and anesthetic interventions were incommensurable. In our opinion, the residual anesthetic and neuromuscular blocking effects might have contributed to the more severe adverse clinical outcomes in patients with postanesthesia falls.

Our study outlined a few extraordinary findings in the occurrence of falls after anesthesia. More postanesthesia falls happened during at the bedside, with the presence of caregivers and after the first uneventful getting off the bed. Consistent with a most recent study (Ikutomo et al. 2015), our study also found that in-hospital falls were often reported during daytime. Patients and the accompanists could become less cautious when the patients moved about in these situations, as these are generally considered as safer scenarios during postoperative care. In addition, we also found that all events of postanesthesia falls occurred at general wards, but not in other special care units, since patients admitted to these units are more seriously ill and are mostly confined to bed after operation. Although these characteristic analyses of falls are descriptive and statistical analysis is not applicable, our study underscores that the preventive strategy for postanesthesia falls should also be comprised to all postoperative periods during the inpatient care (Ikutomo et al. 2015).

We identified four potential independent risk factors for the development of postanesthesia falls from the postanesthesia visit records, namely gender, age, ASA classification, and types of anesthesia techniques. Advanced age has been widely recognized as an independent risk factor for inpatient falls (Marschollek et al. 2012; Oliver et al. 2010). In traumatic patients, male gender is associated with high incidence of in-hospital falls (Brown et al. 2013). The ASA classification is a useful functional assessment tool for the physical status of surgical patients. Higher ASA classes predict the occurrence of falls in the postoperative periods (Church et al. 2011). These common reported factors were included in our analysis. In comparison to subpopulation groups (i.e., trauma and veteran patients) (Church et al. 2011; Brown et al. 2013), our study in the general patient population did not suggest that differences in gender and ASA classification affect the occurrence of falls within 24 h after anesthesia. In our hospital, patients with advanced ASA classes (≥III) were more often admitted to the special medical units for intensive postoperative care and thus might reduce the potential risk of falling during the first postoperative day. Nevertheless, we admit that patients with impaired physical status (high ASA classes) are conceivably more vulnerable to falling in the subsequent hospital care after operation (>24 h). Consistent with the previous reports, our investigation indicated that the incidence proportion of postanesthesia falling is significantly increased in patients with older age (mean age of 65 ± 15 years). Patients of older ages are more susceptible to falls due to multiple interacting intrinsic and extrinsic etiologies, such as comorbidity diseases, postural hypotension, muscle weakness, impaired gait, and presence of environmental hazards in hospital (Rubenstein 2006; Gschwind et al. 2011).

The core objective of this study is to determine the impact of anesthesia in the development of falls after surgical or other invasive interventions in the general population. Poisson statistical analysis clearly indicates that types of anesthesia significantly affect the occurrence of in-hospital falls within 24 h after anesthesia management. Incidence proportion of postanesthesia falls was significantly increased in patients who received neuroaxial block. Although neuroaxial blockade does not seem to impair the balance control (Suarez and Macadar 2008), the recovery of sensory and muscle power is usually unpredictable and the unassisted ambulation is longer than it is assumed (Imarengiaye et al. 2003). Furthermore, the motor and functional recoveries are variable between individual subjects following intrathecal administration of different local anesthetics (Frey et al. 1998). In clinical practice, we recommend that patient ambulation should only be allowed 8 h after regional block, and we do not routinely check the recovery profiles of patients after discharged from the recovery room. Therefore, it could be the main reason that patients might undertake unassisted ambulation without recognizing the incomplete recovery of muscle power of the lower extremities, and thus resulted in falling. We also realize that the three patients who fell after spinal anesthesia were even older (52, 76, and 92 years) than the mean age of all other falling cases (62 years). Furthermore, there was a postoperative fall patient received inhalational anesthesia via laryngeal mask for knee replacement surgery, and single injection of femoral nerve block was applied as an adjunct for perioperative pain management. Although the sample size is too small to draw any conclusive statements, our results are consistent with the previous reports that suggested that regional nerve blockade in older patients could further increase the risk of postoperative falls (Wasserstein et al. 2013). Administration of peripheral nerve block in knee operation has been reported to increase the risk of postoperative falls (Muraskin et al. 2007; Sharma et al. 2010), particularly with the application of continuous lumbar plexus blockade (Johnson et al. 2013). Therefore, any pharmacologic inhibition of nerve conduction in lower extremities following central or peripheral nerve blockade could contribute to the increased risk of postoperative falls. In addition, we detected a significant increased incidence proportion of postoperative falls in patients who received intravenous sedation anesthesia for less invasive procedures, such as endoscopic examination and simple hemorrhoidectomy. These three patients who received intravenous anesthesia were generally less seriously ill (ASA class II-III), and falls happened within 4 h after anesthesia. The reasons for postanesthesia falls in patients received sedation anesthesia are unclear, but these fall events could probably be preventable if higher attention was applied to the postoperative period, even though the anesthesia management was less invasive.

### Limitations of the study

There are several limitations in our study. This is a retrospective case-series chart-review study, and it could therefore be subjected to incomplete collection of all cases throughout the studied period. However, we admit that in-hospital falls has been an imperative monitoring parameter of health care quality assurance in our hospital. Therefore, missing of case record is most unlikely to happen. In addition, prevention of in-hospital falls has been one of the most important medical care issues in our hospital, as the “anti-falling partition board” was established by the medical and nursing staff in 2005. The occurrence of falling has thus significantly reduced after the application of fall prevention programs. Following the introduction of laryngeal mask airway, the use of regional anesthesia has reduced in our hospital due to the improved safety profile with general anesthesia. As a result, the total number of regional anesthesia in our hospital is relatively lower than other institutes. Our postanesthesia visit recorded only the identifications of the patients and did not contain patients’ laboratory data and the perioperative medicine. Hence, we were not able to demonstrate the potential effects of these factors in the occurrence of in-hospital falls after anesthesia. Nevertheless, gender, age, ASA class, and anesthesia techniques are the four most indispensable patient demographical data that should be available in the record of all institutes worldwide. We address that these simple, but basic parameters, may provide useful information in the identification of the high-risk groups of postanesthesia falls.

## Conclusion

The overall incidence proportion of postanesthesia falls is at least 1.6 cases per 10,000 patients over a 24-h observation period. Falls are more commonly happened during the less expected periods after operation and are increased in the elderly and patients who received regional anesthesia. This study highlights that more comprehensive clinical practice guidelines for postoperative care should be exercised to prevent the in-hospital falls.

## Abbreviations

ASA: American Society of Anesthesiologists
IRB: institutional review board
